# An Efficient Ensemble Approach for Alzheimer’s Disease Detection Using an Adaptive Synthetic Technique and Deep Learning

**DOI:** 10.3390/diagnostics13152489

**Published:** 2023-07-26

**Authors:** Muhammad Mujahid, Amjad Rehman, Teg Alam, Faten S. Alamri, Suliman Mohamed Fati, Tanzila Saba

**Affiliations:** 1 Department of Computer Science, Khwaja Fareed University of Engineering and Information Technology, Rahim Yar Khan 64200, Pakistan; mujahidws890@gmail.com; 2 Artificial Intelligence & Data Analytics Lab CCIS, Prince Sultan University, Riyadh 11586, Saudi Arabia; arkhan@psu.edu.sa (A.R.); sgaber@psu.edu.sa (S.M.F.); tsaba@psu.edu.sa (T.S.); 3 Department of Industrial Engineering, College of Engineering, Prince Sattam bin Abdulaziz University, Al Kharj 11942, Saudi Arabia; t.alam@psau.edu.sa; 4 Department of Mathematical Sciences, College of Science, Princess Nourah Bint Abdulrahman University, P.O.Box 84428, Riyadh 11671, Saudi Arabia

**Keywords:** Alzheimer’s disease, ADASYN, deep learning, medical MRI brain images, optimized ensemble model

## Abstract

Alzheimer’s disease is an incurable neurological disorder that leads to a gradual decline in cognitive abilities, but early detection can significantly mitigate symptoms. The automatic diagnosis of Alzheimer’s disease is more important due to the shortage of expert medical staff, because it reduces the burden on medical staff and enhances the results of diagnosis. A detailed analysis of specific brain disorder tissues is required to accurately diagnose the disease via segmented magnetic resonance imaging (MRI). Several studies have used the traditional machine-learning approaches to diagnose the disease from MRI, but manual extracted features are more complex, time-consuming, and require a huge amount of involvement from expert medical staff. The traditional approach does not provide an accurate diagnosis. Deep learning has automatic extraction features and optimizes the training process. The Magnetic Resonance Imaging (MRI) Alzheimer’s disease dataset consists of four classes: mild demented (896 images), moderate demented (64 images), non-demented (3200 images), and very mild demented (2240 images). The dataset is highly imbalanced. Therefore, we used the adaptive synthetic oversampling technique to address this issue. After applying this technique, the dataset was balanced. The ensemble of VGG16 and EfficientNet was used to detect Alzheimer’s disease on both imbalanced and balanced datasets to validate the performance of the models. The proposed method combined the predictions of multiple models to make an ensemble model that learned complex and nuanced patterns from the data. The input and output of both models were concatenated to make an ensemble model and then added to other layers to make a more robust model. In this study, we proposed an ensemble of EfficientNet-B2 and VGG-16 to diagnose the disease at an early stage with the highest accuracy. Experiments were performed on two publicly available datasets. The experimental results showed that the proposed method achieved 97.35% accuracy and 99.64% AUC for multiclass datasets and 97.09% accuracy and 99.59% AUC for binary-class datasets. We evaluated that the proposed method was extremely efficient and provided superior performance on both datasets as compared to previous methods.

## 1. Introduction

Alzheimer’s disease (AD) is an incurable neurological disorder that leads to a gradual decline in cognitive abilities, but early detection can significantly mitigate symptoms [1]. Patients with AD lose their cognitive abilities, making it difficult to carry on with normal responsibilities and perform daily routine task; thus, they become dependent on their family for small tasks and survival. AD causes problems of memory loss like remembering things, arranging and recollecting things, intuition, and judgmental issues [2]. Around 2% of people at the age of 65 are affected with AD and 35% at the age of 85 years. It was reported that 26.6 million people were affected in the year 2006, and the count is increasing dramatically [3]. In 2020, more than 55 million people were affected by AD, and the count is estimated to reach 152 million by 2050 [4]. The degradation of brain cells and the dysfunction of synaptic and pathological changes start to develop almost 20 years before AD diagnosis [5]. A proper diagnosis of the disease is also needed to develop the necessary drugs to slow down the progression process, and the patient’s whole medical history is thoroughly examined for the effective monitoring of the disease. The overall cost and effort faced by patients and families are also increasing dramatically. Researchers have emphasized the importance of the early detection of AD for starting treatment promptly and obtaining accurate results.

Individuals with AD typically exhibit a reduction in brain tissue volume in the hippocampus and cerebral cortex, accompanied by an expansion of the ventricles in the brain, as observed in multiple studies. In advanced stages of the disease, brain scans such as MRI images show a substantial reduction in the hippocampus and cerebral cortex, along with ventricular expansion [6]. AD primarily affects the regions of the brain and the intricate network of brain tissues involved in cognition, memory, decision making, and planning. The diffusion of brain tissues in the affected areas causes a decrease in the MRI image intensities in both the magnetic resonance imaging (MRI) and functional magnetic resonance imaging (fMRI) techniques [7,8,9].

In recent years, there has been a growing trend of using neuroimaging data and machine learning (ML) methods to characterize AD, providing a potential means for personalized diagnosis and prognosis [10,11,12]. Currently, deep learning (DL) has emerged as a powerful methodology in the diagnostic imaging field, as evidenced by several recent studies [13,14,15,16,17]. Diagnosing AD using DL is still a significant challenge for researchers [18]. Medical images are scarce and of lower quality, and the difficulty in identifying regions of interest (ROI) within the brain and unbalanced classes are issues encountered in detecting AD. Among the various DL architectures, the convolutional neural network has received considerable interest due to its extraordinary effectiveness in classification [19]. In contrast to conventional machine learning, deep learning enables automatic feature extraction like low-level to high-level latent representations. Therefore, deep learning requires minimal image pre-processing steps and little prior understanding of the synthesis process [20].

The imbalanced datasets for medical disease detection are the most significant challenge. The number of samples in each class is not equal for Alzheimer’s disease, despite the availability of a balanced dataset. The model’s performance is biased, and generalizations become difficult with imbalanced datasets. Individual deep learning models handle basic data efficiently, but overfitting occurs when dealing with complex problems. The generalizability, efficacy, and reliability of this type of model are poor. Individual deep learning models make predictions or detections based on learning with a single set of weights and do not capture nuances from all image features. To accurately diagnose a disease using segmented magnetic resonance imaging, it is necessary to conduct an in-depth examination of the disease-specific tissues. Several studies have used conventional machine-learning approaches to diagnose diseases from MRI, but manually derived features or the physical examination of medical data and patient records are more complex, time-consuming, and require a significant level of medical staff involvement. The conventional method does not provide a precise diagnosis, resulting in errors during diagnosis and inefficiencies.

Deep learning automates the detection process, making it more efficient and faster. An accurate diagnosis is crucial in cases where early detection is essential for proper treatment. Deep learning models have demonstrated an extraordinary ability to learn nuanced patterns from complex and high-dimensional data. They can automatically extract pertinent information from the images and overcome the limitations of traditional methods. The proposed method addresses the data imbalance issues more efficiently with adaptive synthetic oversampling techniques and makes diagnostics faster. The proposed method combines the predictions of multiple models to make an ensemble and stronger model that learns complex and nuanced patterns from the data. The proposed method is more robust, reliable, and diverse in its decision making. Our objective was to examine the ensemble model’s capacity to detect AD and perform feature extraction in order to improve the model’s overall effectiveness. The following are main contributions of our study:

1. An efficient ensemble approach was proposed that combines VGG16 and Efficient-Net-B2 for Alzheimer disease classification with high accuracy using multiclass and binary-class datasets, also exploring the effect of transfer learning to improve the performance of the model.
2. The adaptive synthetic oversampling technique was applied to a highly imbalanced dataset to balance the Alzheimer’s disease classes. The efficacy of the ADASYN in terms of model overfitting was also investigated to increase the generalization performance of deep learning models.
3. The efficacy of the proposed method was analyzed using k-fold cross-validation and comparing with other state-of-the-art approaches. We also performed a comparison of ensemble and individual deep learning models.

In this paper, we organized our content into several sections. Section 2 presents a comprehensive review of the relevant literature. Section 3 outlines the pre-processing, methods, and performance measures. The results and discussion are presented in Section 4. Section 5 provides the concluding remarks for this paper.

## 2. Literature Review

Due to the prevalence and challenging nature of Alzheimer’s disease (AD), it poses difficulty for experts regarding diagnosis, which has been extensively studied in the literature. The authors of [21], conducted a study in which they utilized Alzheimer’s data to perform a classification process. Their dataset comprised three classes, and they employed Dense-Net as the model, with soft-max serving as the classification layer. The study resulted in an accuracy of 88.9%. While the results were favorable, there remained potential for further improving the accuracy of the model. In addition, Yildirim et al. [22] conducted a study on AD classification using a four-class dataset. They employed convolutional neural network (CNN) architectures and compared the results with their proposed hybrid model, built upon a Resnet50 base and utilizing its knowledge. According to the authors, the hybrid model achieved an accuracy rate of 90%, which outperformed the success rate of pre-trained CNN models. The detection of AD has been extensively researched, and it poses various challenges. The authors of [23] utilized a sparse auto-encoder and 3D CNN to develop a model that could detect disease cases in affected individuals based on the magnetic resonance imaging (MRI) of the brain. The use of three-dimensional convolutions was a significant breakthrough, as it outperformed two-dimensional convolutions. Although the convolution layers were pre-trained with an auto-encoder, they were not fine-tuned, and it was anticipated that fine-tuning would lead to improved performance [24].

Researchers worldwide have shown great interest in classifying AD. The dominant technique for identifying healthy data from fMRI images is to extract features with a CNN, followed by deep learning (DL) classification. The authors of [25] used a deep CNN to classify Alzheimer disease versus normal patients with Alzheimer’s functional MRI data and structural MRI data, achieving 94.79% accuracy with the LeNet5 method and 96.84% accuracy with the Google-Net method. Recently, there has been a notable increase in the use of DL methods in various fields because of their superior performance compared to traditional methods. One study [26] developed a hybrid model that involved using extracted patches from an auto-encoder combined with convolutional layers. Another study [23] improved upon this by incorporating 3D convolution.

In a previous study [27], auto-encoders arranged in a stack with a soft-max layer were used for classification. Another study [28] utilized standard CNN architectures by intelligently selecting training data and utilizing transfer learning but did not achieve remarkable results. A comprehensive comparison was conducted in another study [29], which examined the results and trained data using scratch with fine-tuning. Based on the findings, in most cases, the latter outperformed the former. Fine-tuned CNNs have been used to solve numerous medical imaging problems, including plane localization in ultrasound images [30].

As discussed above, the use of transfer learning (TL) in the medical discipline is significant for detecting AD with sufficient precision. Other research [31] emphasized the use of unsupervised feature learning, which involved two stages. The first stage was to extract features from unprocessed data using two methods—scattered filtering and uncontrolled neural layer networks. To classify healthy and unhealthy individuals, sparse filtering and regression with soft-max were employed. Additionally, some unsupervised learning techniques, including Boltzmann machines and dispersed coding, were used to dispose of the collected data. The ADNI dataset containing cerebrospinal fluid was used in this approach, with a total of 51 AD patients, including 43 with mild signs of AD. MRI scans were collected using 1.5 T scanners. In their study, the authors of [32] proposed a technique that utilized ML algorithms to gather information about a patient’s behavior over time. By employing Estimote Bluetooth beacons, the method accurately determined the location of the patient within the house, with a precision of up to 95%.

Gerardin and team investigated the use of hippocampal texture features [33] as an MRI-based diagnostic tool for early-stage AD, achieving a classification accuracy of 83%. They determined that the hippocampal feature outperformed other techniques in distinguishing stable MCIs and MCI to Alzheimer disease converters. Liu and colleagues [34] used stacked DL auto-encoders with soft-max at the output layer to address the bottleneck issue, achieving a remarkable accuracy of 87.67% for multiclass classification with minimal input data and training. The researchers concluded that combining multiple features would lead to more precise classification results.

The authors of [35] demonstrated the effect of transfer learning on image classification and showed that fine-tuning produced better results. Alzheimer’s disease was diagnosed in [36] employing convolutional-neural-network-based architecture and magnetic resonance brain imaging. The VGG-16 model was deployed as a classification feature extractor. The findings showed that the proposed model for Alzheimer’s disease was 95.7% correct. The study [37] introduced a transfer learning strategy to localize plans in ultrasound scans that could transfer knowledge on fewer layers. Another study [38] proposed an architecture that utilized a transfer learning approach for the detection of Alzheimer’s disease from a multiclass, open-access series of imaging study datasets. The architecture was tested on pre-processed unsegmented and segmented images. The architecture was tested on both binary and multiclass datasets. The results demonstrated that the proposed architecture attained a 92.8% accuracy on multi-class and an 89% accuracy on binary-class datasets.

Iram [39] conducted research on the detection of Alzheimer’s disease using biosignals and the most common machine learning models, which facilitated neurodegenerative disease diagnosis at an early stage. The dataset was imbalanced; to fix the imbalance, oversampling and undersampling techniques were employed, and missing values were addressed. Multiple metrics were employed by the author to evaluate the performance. This study emphasized the significance of machine learning and signal processing in the early identification of life-threatening diseases like Alzheimer’s. Linear and Bayes classifiers were used. Using the Bayes classifier, the author obtained greater accuracy in diagnosis. Kim [40] developed machine learning algorithms for the identification of Alzheimer’s disease biomarkers. The predictive performance of models employing multiple biomarkers was more effective to that of models employing an individual gene.

Biosignals were used by Han et al. [41] to identify dementia in elderly people. They employed no artificial intelligence techniques in their analysis. Insufficient participation made it impossible to derive broad generalizations. A number individuals with moderate dementia should be tested from a broader population. Similar to this, another study [42] employed biosignals to analyze cognitive disorders including Alzheimer’s and Parkinson’s diseases. The authors developed a novel, economical approach for disease identification. Hazarika et al. [43] presented a light-weight, inexpensive, and fast diagnosis method that used brain magnetic resonance scans. They used the DenseNet121 model, which was very expensive and able to detect the disease with 87% accuracy. However, the authors developed and combined two models, AlexNet and LeNet, with fine-tuning. Their method extracted features by utilizing three parallel filters. Their study demonstrated that their model accurately detected the disease with a 93% accuracy rate.

The researchers in [44] used the CNN-based transfer learning architecture VGG-16 to classify Alzheimer’s disease and achieved 95.7% accuracy. Murugan et al. [45] proposed deep learning for dementia and Alzheimr’s disease classification from magnetic resonance images. Several studies in the literature have faced class imbalance issues for Alzheimer’s disease detection because imbalanced datasets lead to overfitting, inaccurate results, and low accuracy among deep learning models. Another problem is that there are not enough data available for training deep learning models. Therefore, we utilized the adaptive synthetic technique (ADASYN), which creates new data samples synthetically, as deep learning models perform best with balanced datasets.

## 3. Proposed Methodology

**Figure 1:**
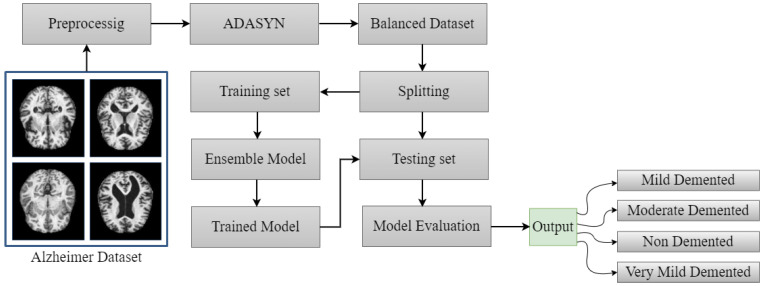
Proposed workflow diagram for Alzheimer’s disease detection.

This section describes the Alzheimer’s disease dataset, pre-processing, adaptive synthetic oversampling technique, deep learning and ensemble models, model evaluation metrics, and classification results. Figure 1 briefly represents the workflow of the proposed method. The pre-processed dataset was then utilized for training the pre-trained and proposed method to efficiently and accurately detect Alzheimer’s disease cases. When the training process was complete, the performance of the models was investigated based on unseen data. In the following subsections, the proposed methodology is discussed.

### 3.1. Dataset Description and Pre-processing

The two Alzheimer’s disease datasets used in this study were collected from Kaggle’s data repository. The multiclass dataset contained four classes, namely mild demented, moderate demented (MD), non-demented (ND), and very mild demented (VMD). A person suffering from the ND class experiences disability in terms of behavioral skills, difficulty in learning and remembering things and the skills of thinking and reasoning, and it even affects the patient’s personal life. However, dementia is not necessarily caused by aging, and its main sign is not memory loss. In the very mild demented (VMD) stage, the patient starts to suffer memory loss, forgetting where he/she put their belongings, recent names they heard, etc. It is hard to find VMD patients through the cognitive capacity test. In the mild demented (MD) phase, the patient is unable to complete their work properly, forgets their home address, and has a hard time remembering things. These patients are not stable and even forget they have memory issues, because they forget everything. This stage is detected by cognitive testing. The fourth class is moderate demented (MOD), which is the most alarming stage because the patient loses their ability to understand anything and faces problems with calculation; it becomes difficult for them to leave home on their own because they forget the way; and they forget important historical events and activities they performed recently.

Table 1 shows the MMSE score and gap between the Alzheimer’s disease classes in the dataset. The mild demented class had a 25.12 MMSE score, the moderate demented class 21.77, the non-demented class 23.50, and the very mild demented class 24.51. The average MMSE mean score for all four classes was 23.72, with a 4.49 standard deviation. The largest gap between Alzheimer’s disease classes was for the mild demented and moderate demented classes at 3. The smallest gap was 0.59 for the mild demented and very mild demented patients.

The images of AD in the dataset were RGB images with different numbers of pixels. The ND class contained 3200 samples, while the MD class contained 896 images, the VMD class contained 2240, and the MOD class contained 64. The only disadvantage of this dataset was that it was imbalanced. To solve this issue, we used ADASYN for class balancing. Another binary MRI Alzheimer’s dataset contains 965 AD and 689 MCI images. Medical image pre-processing is very important to achieve quality results and increase the image quality for machine and deep learning [46]. The images had different heights and widths, and to train the deep learning models, we needed fixed-size inputs. Therefore, we resized all the images to a fixed size of 224 × 224 × 3.

### 3.2. Adaptive Synthetic (ADASYN) Technique

Adaptive synthetic (ADASYN) oversampling technology is used in classification tasks to handle imbalanced classes in datasets. ADASYN creates new synthetic samples from the minority class to address the class imbalance issues. It improves the generalization accuracy of various classifiers. ADASYN is mainly used for object detection, facial expressions, and image analysis to balance the classes. It is a very effective and flexible technique compared to any other oversampling technique. Researchers have utilized the ADASYN oversampling technique to balance an imbalanced dataset for tuberculosis detection from CXR images. They balanced the minority classes with the ADASYN technique to enhance the overall effectiveness of the tuberculosis detection model and achieved a high accuracy compared to other techniques [47]. Table 2 shows the training and testing images after splitting the balanced data. Algorithm 1 shows the steps of the ADASYN technique.
**Algorithm 1** ADASYN algorithm**Input:** Data_images ⟵ *Input features*Labels ⟵ *Corresponding labels***Output:** X_res ⟵ *Oversampled image f eature vectors*y_res ⟵ *Oversampled corresponding labels***Start:**        1: *Import ADASYN f rom imblearn.over_sampling*    2: *Create ADASYN oversamping instance and assign a variable sm*    3: *Applying the ADASYN oversampling technique to the dataset*    4: *Fit resample method is called and passes through the arguments* (*Data_images and Labels*).    5: *Assign Oversampled image f eature vectors to X_res*    6: *Assign Oversampled corresponding labels to y_res*    7: *Return* (*X_res*, *y_res*)**End**

### 3.3. Ensemble Deep Learning with Transfer Learning Approach

Typically, constructing a deep learning architecture is a challenging task. The weights that one uses in deep learning are allocated before the training phase and changed continuously. Deep learning requires a lot of time to change the weights repeatedly, which leads to the overfitting of the model. Transfer learning (TL) has been the most effective method to overcome the aforementioned problems [48]. Transfer learning leverages previously learned knowledge from pre-trained models trained on large datasets. In addition, it adjusts the hyper-parameters and tunes the hidden layers of pre-trained models. The efficiency of deep learning may be improved by TL, which helps to save time and effort [49].

Ensemble learning is the most essential approach for improving the overall performance of several individual deep learning models. Ensemble learning trains many deep learning models on the same datasets and integrates them so effectively that the predictions made by the models are accurate and the detection accuracy increases [50]. Ensemble learning may be applied in a variety of medical diagnosis tasks. Overall, it improves performance, makes models more robust, and reduces the chances of overfitting. By combining the aspects of several models, deep learning can learn simple and complex patterns efficiently. Five ensemble deep models were used in this Alzheimer’s disease detection study to efficiently detect cases of Alzheimer’s disease from multiclass and binary-class classification datasets. The input layers, output shape, and parameters of the proposed ensemble model are presented in Table 3.

**Figure 2:**
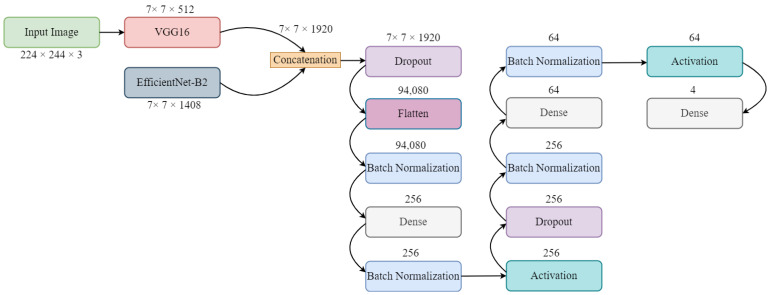
Architecture of proposed ensemble model.

The proposed ensemble deep learning model is shown in Figure 2. Firstly, we imported the VGG-16 and EfficientNet-B2 models from the keras application and other important libraries relevant to the model. The input image shape for the ensemble model was 224 × 224 × 3. Then, we loaded both the pre-trained deep learning models with include-top equal to false (without top layers). The input shape for the ensemble models was created and kept the same. After that, we concatenated the output of both the VGG-16 and EfficientNet-B2 models using the “concatenate” function. A dropout layer was added immediately after the concatenation layers. The flatten layers function was used to convert the features into a specific format that was acceptable for the fully connected layer. We then fine-tuned the other layers to accelerate the training steps and increase the overall progress. Four batch normalizations and three dense layers were used with activation functions. Batch normalization is a very popular method that normalizes layers as well as providing stability to neural networks. It also makes learning easier and faster. The testing accuracy may be improved with batch normalization, depending on the type of data. Dense layers are regularly used for image classification. Finally, the model was compiled with the “Categorical Cross-entropy” loss function and Adam optimizer.

### 3.4. Fine-Tuned Individual Deep Learning Models

This subsection covers a brief description of certain deep learning (DL) models, namely convolutional neural networks (CNNs), DenseNet121, VGG16, Xception, and EfficientNet-B2. It also analyzes the performance of the trained model using performance metrics like accuracy, AUC, recall, precision, and F1 score.

#### 3.4.1. CNN

CNNs are considered the most significant DL models. Unlike traditional matrix multiplication, CNNs employ convolution in their operation. Their primary application is in object classification using image data. CNNs are a type of deep learning model that are widely used for image and video processing tasks. The structure and function of the visual cortex in the brain inspired these networks. A CNN’s operation involves several processing layers, including convolutional layers, pooling layers, and fully connected layers. Overall, CNNs are powerful tools for pre-processing tasks and have been used for various applications, including object detection, facial recognition, and autonomous driving [51,52].

**Figure 3:**
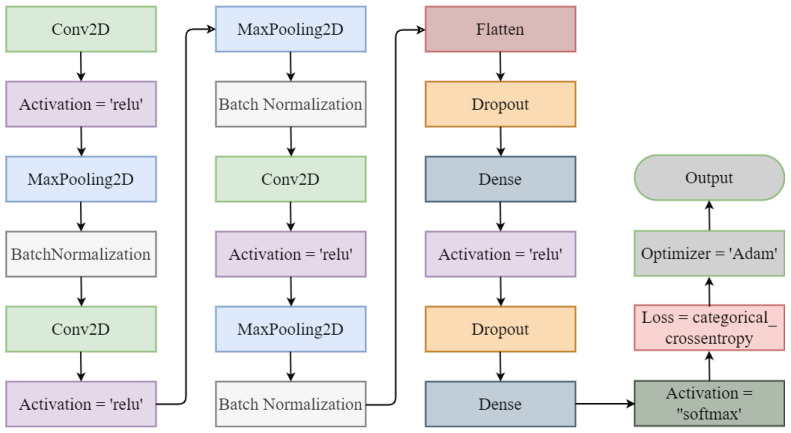
Convolutional neural network architecture.

The CNN architecture is shown in Figure 3. It took an input size of 224 × 224 × 3. The CNN architecture had three convolutional two-dimensional layers followed by the RelU activation function, three max pooling layers, and three batch normalization layers. Then, a flattening layer was added to follow the dropout layer. Two dense layers were included, one followed by the activation of the ’RelU’ function and the other by the activation of the soft-max layer.

#### 3.4.2. DenseNet121

DenseNet121 [53] is a CNN architecture that has been commonly employed for image classification tasks. It was introduced in 2017 as an improvement upon the previous popular architectures such as VGG and ResNet. DenseNet121 employs a dense connectivity pattern, where each layer receives feature maps from all previous layers and passes its feature maps to all successive layers. This dense connectivity allows for better gradient flow and parameter efficiency and reduced vanishing gradient problems. The architecture has 121 layers, including convolutional, pooling, and dense blocks, and has achieved state-of-the-art performance on several benchmark datasets such as ImageNet.

#### 3.4.3. EfficientNet-B2

EfficientNetB2 is a CNN architecture that is part of the EfficientNet family of models. It was designed to provide an optimal balance between model size and performance for image classification tasks. EfficientNetB2 is larger and more complex than the original EfficientNetB0 model, but it maintains the same basic structure, including the use of compound scaling to balance depth, width, and resolution. EfficientNetB2 has 7.8 million parameters. It is often used as a baseline model for transfer learning or fine-tuning specific image classification tasks [54].

#### 3.4.4. VGG16

VGG-16 is a deep CNN architecture that was developed by the visual geometry group (VGG) at the university of Oxford in 2014. It is a widely used model for image recognition tasks and has achieved state-of-the-art results in many computer vision (CV) benchmarks. The architecture of VGG16 contains 16 layers, including 13 convolutional layers and 3 fully-connected layers. The convolutional layers have small 3 × 3 filters and are placed on top of each other, increasing the depth of the network. The use of small filters with a small stride size helps preserve spatial information and enables the network to learn more complex features [55].

#### 3.4.5. Xception

Xception is a deep CNN architecture that was proposed in 2016. It was inspired by the inception architecture but differs from it by replacing the standard convolutional layers with depth-wise separable convolutions. This approach minimizes the number of training parameters and computations, resulting in faster and more efficient training. Xception also employs skip connections to allow for better gradient flow and improved accuracy. The architecture has achieved state-of-the-art results on various image classification benchmarks such as ImageNet, and it has been widely used in computer vision applications [56].

### 3.5. Performance Measures

Evaluation metrics are quantitative measures used to assess the performance of a model or system in solving a specific task. The model’s classification results could be divided into four classes: true-positive (TP), true-negative (TN), false-positive (FP), and false-negative (FN). TP refers to correctly identified positive instances, while TN refers to accurately identified negative instances. FP represents falsely predicted positive instances, and FN represents falsely predicted negative instances. Various evaluation parameters were utilized in this study, including recall, precision, accuracy, AUC, and F1 score.

(1) $$\mathrm{Accuracy}=\frac{\mathrm{TP}+\mathrm{TN}}{\mathrm{TP}+\mathrm{TN}+\mathrm{FP}+\mathrm{FN}}*100$$

(2) $$\mathrm{Precision}=\frac{\mathrm{TP}}{\mathrm{TP}+\mathrm{FP}}*100$$

(3) $$\mathrm{Recall}=\frac{\mathrm{TP}}{\mathrm{TP}+\mathrm{FP}}*100$$

(4) $$F1-\mathrm{score}=2*\frac{\mathrm{pre}+\mathrm{Rec}}{\mathrm{Pre}*\mathrm{Rec}}*100$$

(5) AUC=0.5∗(TPR+TNR)∗100

## 4. Results and Discussion

Experiments were conducted using a Hewlett Packard Core i5, sixth-generation, 25 GB RAM, and a colab Pro GPU that was manufactured by Google were used in this study. This section presents all the experiments conducted on the binary and multiclass Alzheimer’s brain disease datasets. We utilized efficient ensemble deep learning architectures that consumed minimum resources. We utilized a 32-bit batch size, 15 epochs, a learning rate of 0.0001, a cross-entropy loss function, Adam, and an SGD optimizer.

### 4.1. Results of Individual Fine-Tuned Deep Learning Models

Experiments were conducted using individual fine-tuned deep learning models including VGG-16, DenseNet-121, EfficientNet-B2, CNN, and Xception. These individual models were trained and tested using a loss function named categorical cross-entropy for mild demented, moderate demented, non-demented, and very mild demented cases and an Adam optimizer to optimize the performance. A batch normalization layer was added to Efficient-Net-B2, Xception, and VGG-16 to increase the training process, reduce the learning time, and lower the generalization errors. Moreover, a dropout layer was utilized to avoid overfitting. There were 50 epochs implemented for each model. Table 4 presents the results of the individual pre-trained models. For the individual models, DenseNet-121 attained the lowest accuracy, precision, recall, F1 score, and area under the curve for Alzheimer’s disease multiclass classification. The second most poorly performing deep model was Xception, which achieved a 75.04% accuracy and 93.70% area under the curve. Both the CNN and VGG-16 models achieved almost the same classification accuracy. The fine-tuned high-performance model EfficientNet-B2 achieved a 95.89% accuracy and 95.95% recall score. EfficientNet-B2 performed well in individual deep learning models.

**Figure 4:**
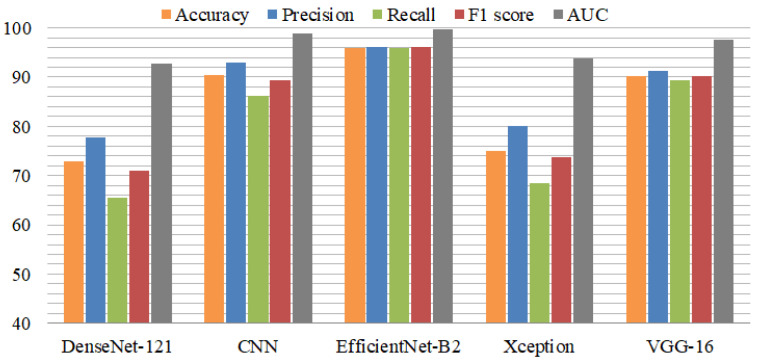
Comparison of individual models using performance metrics.

Figure 4 shows the performance comparison of individual models using various metrics. DenseNet-121 and Xception performed poorly in terms of recall score and F1 score. EfficientNet-B2 performed exceptionally, in addition to VGG-16. The area under the curve (AUC) was better than the other metrics.

### 4.2. Results of Ensemble Deep Learning Models with Multiclass Dataset

The ensemble deep learning model results are presented in Table 5. The ensemble EfficientNet-B2 and DenseNet-121 model achieved a 96.96% accuracy, 97% precision, 96.98% recall, 96.93% F1 score, and 99.60% area under the curve (AUC) score. The second VGG-16-DenseNet-121 ensemble model achieved a 95.56% accuracy and 98.75% AUC. The EfficientNet-B2+Xception model achieved a 96.26% accuracy, 96.50% recall, and 99.11% AUC. Xception+DenseNet-121 achieved a 91.05% accuracy. The proposed VGG-16+EfficientNet-B2 model achieved a 97.35% accuracy score and a 99.64% area under the curve (AUC). All the ensemble models performed well and accurately detected the AD cases from the multiclass dataset. The DenseNet-121+Xception ensemble model achieved an 18% higher accuracy than the individual DenseNet-121 and Xception models. The other ensemble model achieved 1.46% better results when we compared it with the individual EfficientNet-B2 model.

**Figure 5:**
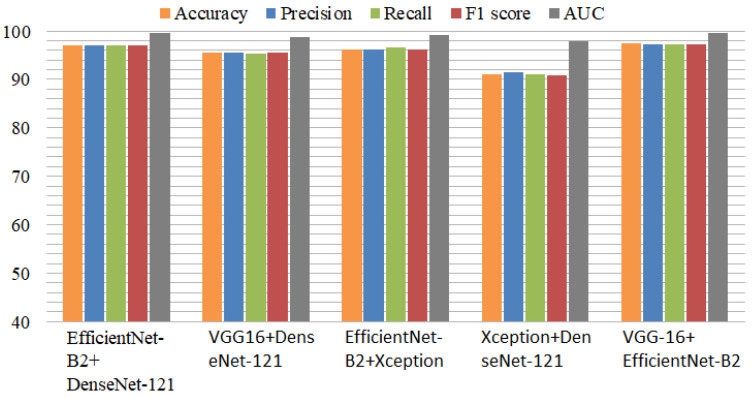
Comparison of ensemble deep models using performance metrics with balanced dataset.

The performance comparison of the ensemble models is presented in Figure 5. Among the ensemble models, VGG-16+EfficientNet-B2 performed efficiently, with high performance metrics. The Xception model with Efficient-Net-B2 provided better results than the individual Xception model. Similarly, DenseNet-121 with VGG-16 performed with high accuracy for detecting Alzheimer’s disease. The experiments proved that the ensemble models provided excellent results compared to the individual models in terms of all performance metrics.

The results of the ensemble deep learning models using the imbalanced dataset are shown in Table 6. The ensemble model of EfficientNet-B2 and DenseNet-121 obtained an accuracy of 92.82%, a precision of 94.29%, a recall of 93.76%, an F1 score of 91.52%, and an area-under-the-curve (AUC) score of 99.38%. The second ensemble model of VGG-16-DenseNet-121 had an accuracy of 91.52% and an AUC of 98.98%. The EfficientNet-B2+Xception model had an accuracy of 90.45%, a recall of 87.80%, and an AUC of 98.80%. Xception+DenseNet-121 obtained an accuracy of 89.29%. The proposed VGG-16+EfficientNet-B2 model obtained an accuracy score of 95% and an AUC of 99.41%. All ensemble models achieved outstanding performance and accurately identified AD cases in the multiclass datasets. Using the imbalanced dataset, the DenseNet-121+Xception ensemble model achieved an 8% lower accuracy. The accuracy of another ensemble model was 7% lower when compared to the balanced dataset.

**Figure 6:**
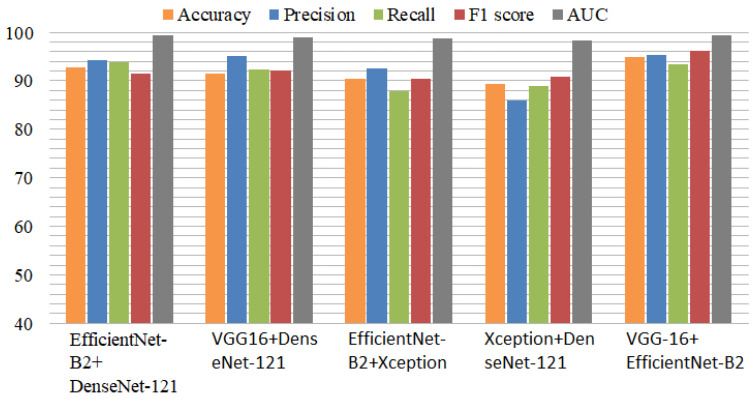
Comparison of ensemble deep models using performance metrics with imbalanced dataset.

Figure 6 displays the performance comparison of the ensemble models using the imbalanced dataset. Among the ensemble models, VGG-16+EfficientNet-B2 performed effectively, with high performance metrics. In comparison to previous models, the DenseNet-121 model with Efficient-Net-B2 offered superior results. In the same way, DenseNet-121 with VGG-16 showed good performance in identifying Alzheimer’s disease. The results showed that the ensemble models with an unbalanced dataset also produced better results. The experiments, however, showed that the proposed approach achieved 2.35% higher accuracy when utilizing the balanced dataset.

Table 7 presents the results of the proposed model with different learning rates to check the impact of the learning rates on the model performance. During the training phase, it was essential to select the appropriate learning rate in order to ensure that the model weights were properly updated. We achieved a 94.47% accuracy and 98.53% AUC by utilizing a 0.01 learning rate. In another experiment, the learning rate was set to 0.001, and a 97.30% accuracy was achieved. When the learning rate was set to 0.0001, we attained a model accuracy of 97.35% and a 99.64% AUC.

**Figure 7:**
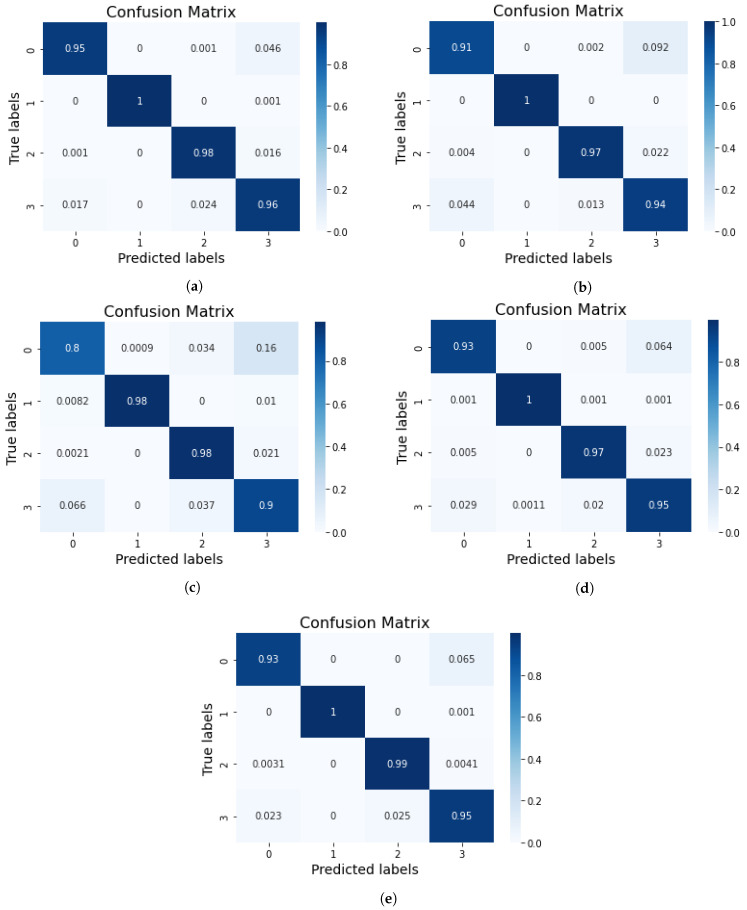
Results of confusion matrix for (**a**) VGG-16+EfficientNet-B2, (**b**) VGG-16+DenseNet-121, (**c**) Xception+DenseNet-121, (**d**) Xception+EfficientNet-B2, and (**e**) EfficientNet-B2+DenseNet-121.

The confusion matrix results of the ensemble deep learning models are shown in Figure 7, where label 0 indicates moderate demented, label 1 indicates non-demented, label 2 indicates mild demented, and label 3 indicates very mild demented. The VGG-16+EfficientNet-B2 model produced 100% true predictions for non-demented cases. The Xception+DenseNet-121 model produced 98% true predictions for non-demented and mild demented Alzheimer’s cases. The Exception+EfficientNet-B2 model also produced the same 100% true predictions for non-demented case. The VGG-16+DenseNet-121 model achieved 91% true predictions for the moderate demented class. The results hence showed that the VGG-16+EfficientNet-B2 model predictions were very good.

**Figure 8:**
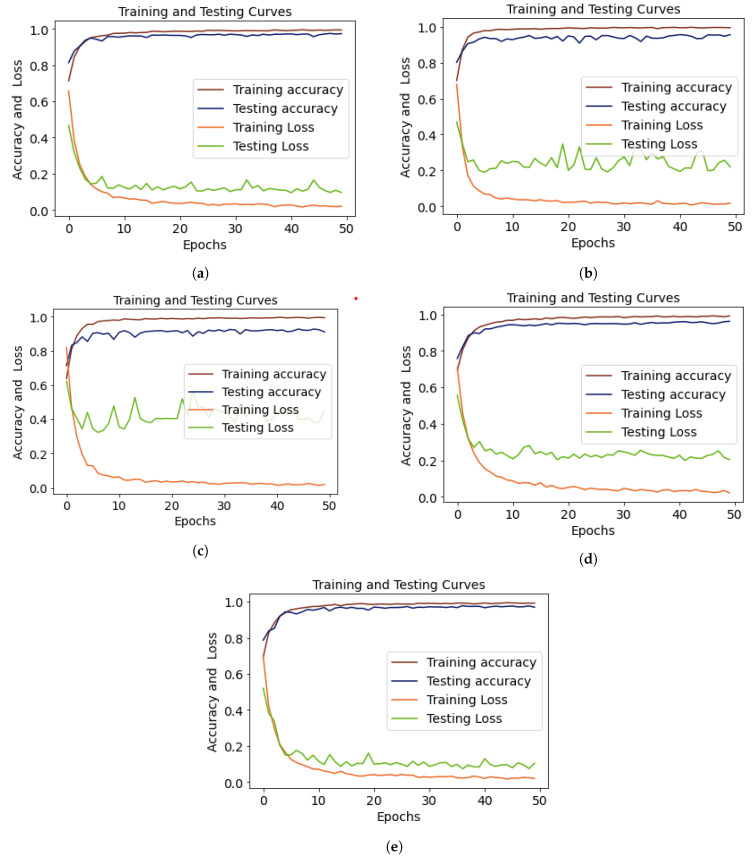
Training and testing curves of (**a**) VGG-16+EfficientNet-B2, (**b**) VGG-16+DenseNet-121, (**c**) Xception+DenseNet-121, (**d**) Xception+EfficientNet-B2, and (**e**) EfficientNet-B2+DenseNet-121, showing accuracy and loss.

The training-testing accuracy and loss are displayed in Figure 8a. We observed that the training accuracy was 81.34 at epoch 1, and by epoch 10, we started to see variations in the data. We chose to train the ensemble deep learning models for 50 epochs, and we were able to improve their performance. Figure 8b shows the performance curves of the ensemble EfficientNet-B2+DenseNet-121 model, where the training accuracy was at its highest point at epoch 45 and the testing accuracy at epoch 37. The testing loss gradually decreased from epoch 1 to epoch 50. The testing loss for the ensemble VGG-16+DenseNet-121 model is displayed in Figure 8c. The curves of the testing and training accuracy started increasing from epoch 1. At epoch 43, the training accuracy reached 99.73 and the loss decreased from 0.68 to 0.01. Figure 8d,e shows that the testing loss for the ensembles of Xception+DenseNet-121 and Exception+EfficientNet-B2 was high compared to that in Figure 8a,b.

### 4.3. Results of Ensemble Deep Learning Models with Binary-Class Dataset

The results of the ensemble models were also evaluated on the binary-class Alzheimer’s disease dataset to test the effectiveness of the proposed model, as shown in Table 8. The EfficientNet-B2+DenseNet-121 model achieved a 95.45% accuracy, 95.10% precision, 95.45% recall, 95.50% F1 score, and 98.68% area-under-the-curve (AUC) score. The second ensemble VGG-16+DenseNet-121 model achieved a 94.90% accuracy and 98.43% AUC. The EfficientNet-B2+Xception model achieved a 91.80% accuracy, 91.80% recall, and 97.34% AUC. The Xception+DenseNet-121 model achieved a 91.05% accuracy. The proposed VGG-16+EfficientNet-B2 model achieved a 97.07% accuracy score and 99.59% area under the curve (AUC). All the ensemble models performed outstandingly and accurately detected the AD cases for the binary-class dataset. The proposed ensemble model also achieved a remarkable 97.07% accuracy on the binary-class classification dataset.

### 4.4. K-Fold Cross-Validation Results for Ensemble Models

The performance and feasibility of the proposed ensemble model were also evaluated with k-fold cross-validation. The results of the cross-validation are displayed in Table 9. The experiments validated that with k-fold cross-validation, the performance was also outstanding. The VGG-16+DenseNet-121 model achieved an accuracy score of 0.942 with a +/− 0.02 standard deviation. EfficientNet-B2+ DenseNet-121 achieved an accuracy score of 0.961 with a +/− 0.04 standard deviation. VGG-16+ EfficientNet-B2 achieved a 0.963 accuracy and a +/− 0.03 standard deviation. The results suggested that the proposed ensemble model was fit and accurate enough to detect Alzheimer’s disease from the multiclass MRI image dataset.

### 4.5. Comparison of Proposed Ensemble Model with Previous Studies

To show the effectiveness and robustness of the proposed ensemble model, we performed a comparison of the proposed method with previous studies discussed in related work. Table 10 depicts the results comparison for the detection of Alzheimer’s disease cases. We chose those studies from the literature that considered multiclass datasets for the comparison with the proposed method. Jain et al. [39] proposed convolutional neural networks for AD classification using multiclass images with 95.73% accuracy. Similarly, another researcher [42] used the CNN-based transfer learning architecture VGG-16 to classify Alzheimer’s disease and achieved 95.70% accuracy. Yildirim et al. [23] employed hybrid deep CNN models using a multilclass Alzheimer’s dataset and attained 90% accuracy. Liu et al. [22] utilized a multi-deep CNN for automatic Alzheimer’s disease classification with the lowest accuracy. The results shown in the comparison table were not satisfactory due to the low accuracy and the fact that the models were not properly utilized to achieve outstanding results. However, our proposed ensemble model classified Alzheimer’s disease with the highest accuracy and was more efficient than any other individual or previous pre-trained models.

## 5. Conclusions

The timely diagnosis and classification of Alzheimer’s disease using multiclass datasets is a difficult task. To detect and treat the disease, an accurate automatic system is required. This study proposed a deep ensemble model with transfer learning techniques to detect Alzheimer’s disease cases from a multiclass dataset. The Alzheimer disease dataset was highly imbalanced, and we used adaptive synthetic oversampling (ADASYN) to balance the classes. The proposed model achieved an accuracy of 97.35% in detecting disease cases. The DenseNet-121+Xception ensemble model achieved an 18% higher accuracy than the individual DenseNet-121 and Xception models. Another ensemble model achieved 1.46% better results when we compared it with individual EfficientNet-B2. Our proposed ensemble model was less time-consuming, more efficient, worked well even on small datasets, and did not use any hand-crafted features. The deep learning automatically extracted relevant and key features from the samples, and an ensemble of deep learning models captured various aspects of the given samples in depth. In the future, we will collect and evaluate larger amounts of data to quickly and precisely diagnose Alzheimer’s cases and combine various types of data to enhance the accuracy of detecting models.

## Figures and Tables

**Table 1:** MMSE scores and gaps between the classes in the dataset.

Class	Mean MMSE Score	Standard Deviation of MMSE Score	Gap between the Classes
Mild demented	25.12	4.90	Gap between mild demented and moderate demented: 3.33
Moderate demented	21.77	2.67	Gap between mild demented and non-demented: 1.62
Non-demented	23.50	5.10	Gap between mild demented and very mild demented: 0.59
Very mild demented	24.51	5.28	Gap between moderate demented and non-demented: 1.72
Mean MMSE score	23.72		Gap between moderate demented and very mild demented: 2.72
Mean standard deviation		4.49	Gap between non-demented and very mild demented: 1.01

**Table 2:** Training and testing images after implementing the ADASYN technique.

	MD	MOD	ND	VMD	Total
Training images	2256	2253	2252	2231	8992
Testing images	987	956	958	953	3854
Total	3243	3209	3210	3184	12,846

**Table 3:** Total layers, output shape, and parameters of ensemble model.

Sr #	Layers	Output Shape	Parameters #
1	Input layers	224 × 224 × 3	0
2	VGG-16	7 × 7 × 512	14,714,688
3	EfficientNet-B2	7 × 7 × 1408	7,768,569
4	Concatenate	7 × 7 × 1920	0
5	Dropout	7 × 7 × 1920	0
6	Flatten	94,080	0
7	Batch normalization	94,080	376,320
8	Dense layer	256	24,084,736
9	Batch normalization	256	1024
10	Activation layer	256	0
11	Dropout	256	0
12	Batch normalization	256	1024
13	Dense layer	64	16,448
14	Batch normalization	64	256
15	Activation layer	64	0
16	Dense layer	4	260

**Table 4:** Results of individual fine-tuned deep learning models.

Model	Accuracy	Precision	Recall	F1 Score	AUC
DenseNet-121	72.94	77.74	65.54	70.89	92.69
CNN	90.50	92.87	86.14	89.35	98.84
EfficientNet-B2	95.89	96.18	95.95	96.02	99.72
Xception	75.04	79.99	68.37	73.84	93.70
VGG-16	90.11	91.27	89.26	90.23	97.59

**Table 5:** Results of ensemble deep learning models with multiclass dataset.

Model	Accuracy	Precision	Recall	F1 Score	AUC
EfficientNet-B2+DenseNet-121	96.96	97.00	96.98	96.93	99.60
VGG-16+DenseNet-121	95.56	95.50	95.23	95.50	98.75
EfficientNet-B2+Xception	96.26	96.24	96.50	96.25	99.11
Xception+DenseNet-121	91.05	91.50	91.00	90.75	97.78
VGG-16+EfficientNet-B2	97.35	97.32	97.35	97.37	99.64

**Table 6:** Results of ensemble deep learning models with multiclass imbalanced dataset.

Model	Accuracy	Precision	Recall	F1 Score	AUC
EfficientNet-B2+DenseNet-121	92.82	94.29	93.76	91.52	99.38
VGG-16+DenseNet-121	91.52	95.21	92.43	92.11	98.96
EfficientNet-B2+Xception	90.45	92.61	87.80	90.40	98.80
Xception+DenseNet-121	89.29	85.83	88.90	90.87	98.28
VGG-16+EfficientNet-B2	95.00	95.23	93.34	96.13	99.41

**Table 7:** Results of proposed model with different learning rates.

Accuracy	Precision	Recall	F1 Score	AUC	Learning Rate
94.47	94.55	94.45	94.48	98.53	0.01
97.33	97.30	97.35	97.30	99.60	0.001
97.35	97.32	97.35	97.37	99.64	0.0001

**Table 8:** Results of ensemble deep learning models with binary-class dataset.

Model	Accuracy	Precision	Recall	F1 Score	AUC
EfficientNet-B2+DenseNet-121	95.45	95.10	95.45	95.50	98.68
VGG-16+DenseNet-121	94.90	94.56	94.90	94.97	98.43
EfficientNet-B2+Xception	91.80	91.80	91.80	92.19	97.34
Xception+DenseNet-121	90.53	90.84	90.35	91.04	96.22
VGG-16+EfficientNet-B2	97.07	96.91	97.27	97.16	99.59

**Table 9:** K-fold cross-validation results for proposed models.

Model	Accuracy	Standard Deviation (std)
EfficientNet-B2+DenseNet-121	96.1%	+/− 0.04
VGG-16+DenseNet-121	94.2%	+/− 0.02
EfficientNet-B2+Xception	94.5%	+/− 0.03
Xception+DenseNet-121	91.1%	+/− 0.03
VGG-16+EfficientNet-B2	96.3%	+/− 0.03

**Table 10:** Comparison of proposed ensemble model with previous studies.

Reference	Model	Dataset	Accuracy
[39]	VGG-16	Multiclass	95.73%
[22]	Multi-deep CNN	Multiclass	88.9%
[23]	Deep hybrid model	Multiclass	90%
[24]	3D CNN model	Multiclass	89.47%
[41]	AlexNet	Multiclass	92.85%
[42]	VGG-16	Multiclass	95.70%
[43]	DEMNET	Multiclass	95.23%
**This paper**	**Proposed method**	**Multiclass**	**97.35%**

## Data Availability

Not applicable.
